# *Bifidobacterium infantis* Potentially Alleviates Shrimp Tropomyosin-Induced Allergy by Tolerogenic Dendritic Cell-Dependent Induction of Regulatory T Cells and Alterations in Gut Microbiota

**DOI:** 10.3389/fimmu.2017.01536

**Published:** 2017-11-10

**Authors:** Linglin Fu, Jinyu Song, Chong Wang, Shujie Fu, Yanbo Wang

**Affiliations:** ^1^Food Safety Key Laboratory of Zhejiang Province, School of Food Science and Biotechnology, Zhejiang Gongshang University, Hangzhou, China; ^2^Laboratory of Mucosal Immunology and Food Research, School of Food Science and Biotechnology, Zhejiang Gongshang University, Hangzhou, China

**Keywords:** tropomyosin, allergy, probiotic, dendritic cell, regulatory T cell, microbiota

## Abstract

Shellfish is one of the major allergen sources worldwide, and tropomyosin (Tm) is the predominant allergic protein in shellfish. Probiotics has been appreciated for its beneficial effects on the host, including anti-allergic and anti-inflammatory effects, although the underlying mechanisms were not fully understood. In this study, oral administration of probiotic strain *Bifidobacterium infantis* 14.518 (Binf) effectively suppressed Tm-induced allergic response in a mouse model by both preventive and therapeutic strategies. Further results showed that Binf stimulated dendritic cells (DCs) maturation and CD103^+^ tolerogenic DCs accumulation in gut-associated lymphoid tissue, which subsequently induced regulatory T cells differentiation for suppressing Th2-biased response. We also found that Binf regulates the alterations of gut microbiota composition. Specifically, the increase of *Dorea* and decrease of *Ralstonia* is highly correlated with Th2/Treg ratio and may contribute to alleviating Tm-induced allergic responses. Our findings provide molecular insight into the application of Binf in alleviating food allergy and even gut immune homeostasis.

## Introduction

Food allergy is now a major public health issue, which torments 2% of the adult and 2–8% of children all over the world (1) and presents a remarkable increasing incidence in recent years (2). The most recent prevalence data from Asia highlighted seafood as a significant sensitizer in up to 40% of children and 33% of adults (3), and 0.5–2.5% of the general population are suffering from shellfish allergy (4). Shrimp tropomyosin (Tm) was the first identified shellfish allergen (5–7), and later demonstrated to be a panallergen among variety kinds of invertebrates such as crustaceans, mollusks, insects, arachnids, and helminths (8, 9).

The mucosal surface is the largest area of the body and covers several hundred square meters in an adult, forming the first line of defense as well as the primary route of exposure to antigens. Particularly, gut-associated lymphoid tissue (GALT), a part of mucosal immune system, is the major tissue responsible for allergic response stimulated through oral delivery. In the GALT, sundry immune cells and cytokines participate in and orchestrate the final immune responses such as anaphylactic symptoms or immune tolerance. In the process of food allergy, naïve T cells preferentially differentiate into T helper (Th) cells Th2, which further induce IgE-producing plasma cells, and finally in turn, resulting in mast cells degranulation and histamine releasing. Alternatively, in the process of immune tolerance, naïve T cells are mostly differentiated into Th1 cells so as to inhibit Th2 polarization, or differentiated into regulatory T cells (Tregs), which shut down the overall immune response to oral antigens (10). Additionally, it is believed that dendritic cells (DCs) play the critical role in the selection of the above immune responsive directions by determining the way of naïve T cells differentiation (11). CD103-expressing DCs (CD103^+^ DCs) are present at high frequency in the small intestine and migrate to the mesenteric lymph node (MLN) to initiate oral tolerance (12, 13).

Probiotics are defined as live microorganisms that have a positive effect on the health of the host when administered in adequate amounts (14). Recently, experimental and clinical evidence have shown that probiotics can regulate host immune system or gut microbiota composition, resulting in the effective alleviation of allergic responses (15–24). However, results of clinical studies on the efficacy of prophylactic or therapeutic treatments with different bacterial strains in the context of allergic sensitization have been conflicting (25–27), and the anti-allergic effects of probiotic bacteria are still not completely defined. Thus, many questions remain unanswered, such as which probiotic strains are the most effective in modulation of allergic responses and how orally administrated probiotics affect the systemic immune system (28).

In the present work, we compared the anti-allergic effect of probiotic strain *Bifidobacterium infantis* 14.518 (Binf) in both prophylactic and therapeutic treatments. We also investigated the role of intestinal DCs in the generation of anti-allergic Tregs and in suppressing of Th2-biased allergic responses. Moreover, our seafood allergy animal model reflects that the change of gut microbiota composition is tightly correlated with Th2/Treg balance. Our results provide new evidence of probiotic strain Binf application in food allergy, and also reveal the important role for intestinal DCs and distinct microbiota composition on induction of Tregs after exposure to Tm in the regulation of mucosal Th2 responses to food antigens and intestinal allergic reactivity.

## Materials and Methods

### Ethics Statement

This study was carried out in strict accordance with the recommendations in the National Guide for the Care and Use of Laboratory Animals of China. All animal procedures were approved by the Zhejiang Gongshang University Laboratory Animal Welfare Ethics Review Committee.

### Animals

Six- to-eight-week-old female BALB/c mice were purchased from Laboratory Animal Center of Hangzhou Normal University (Hangzhou, China). Mice are housed in a room with a 12-h light–dark cycle at 22–24°C under specific pathogen-free conditions.

### Bacterial Preparation

The potential probiotic strain of *Bifidobacterium infantis* 14.518 (Binf) that originally obtained from infant fecal samples was obtained from CGMCC (China General Microbiological Culture Collection Center). Binf was routinely grown in MRS medium at 37°C overnight. Bacterial cells were collected by centrifugation at 4,000 rpm for 10 min, suspended in aseptic phosphate-buffered saline (PBS) and adjusted to 2 × 10^9^ CFU/ml. For *in vitro* experiments, the concentration of Binf was adjusted to 10^7^ CFU/ml in PBS and heat treated at 60°C for 30 min to suppress uncontrollable bacterial growth in cell culture medium.

### Shrimp Tropomyosin

The preparation of Tm was carried out as described by Kunimoto et al. (29) with some modifications. Briefly, fresh whiteleg shrimp (*Litopenaeus vannamei*) was minced and homogenized in two volumes of extraction buffer A (50 mmol/l KC1, 2 mmol/l NaHCO_3_). The homogenate was centrifuged at 7,000 *g* for 10 min at 4°C. The precipitate was collected and suspended in two volumes of extraction buffer A. After four repeated cycles of homogenization and centrifugation, the resulting precipitate was dissolved in acetone and passed through a gauze filter. The precipitate was washed with four volumes of acetone and dried overnight at room temperature. The powder obtained was suspended in extraction buffer B (20 mmol/l Tris–Cl, pH 7.5; 1 mol/l KCl; 0.1 mmol/l dithiothreitol) and stirred for 5 days at 4°C. The extract was treated with boiling water bath for 10 min and clarified by centrifugation at 7,000 *g* for 10 min at 4°C. The supernatant was partially purified by ammonium sulfate fractionation at 30% saturation. The pellet was collected by centrifugation at 7,000 *g* for 10 min at 4°C and dissolved in PBS. The protein concentration was determined by using a bicinchoninic acid assay (BCA) kit (Pierce Biotechnology Inc., Rockford, IL, USA) and the purity was analyzed by SDS-PAGE (Figure S1 in Supplementary Material).

### Immunization Protocols

**Figure 1:**
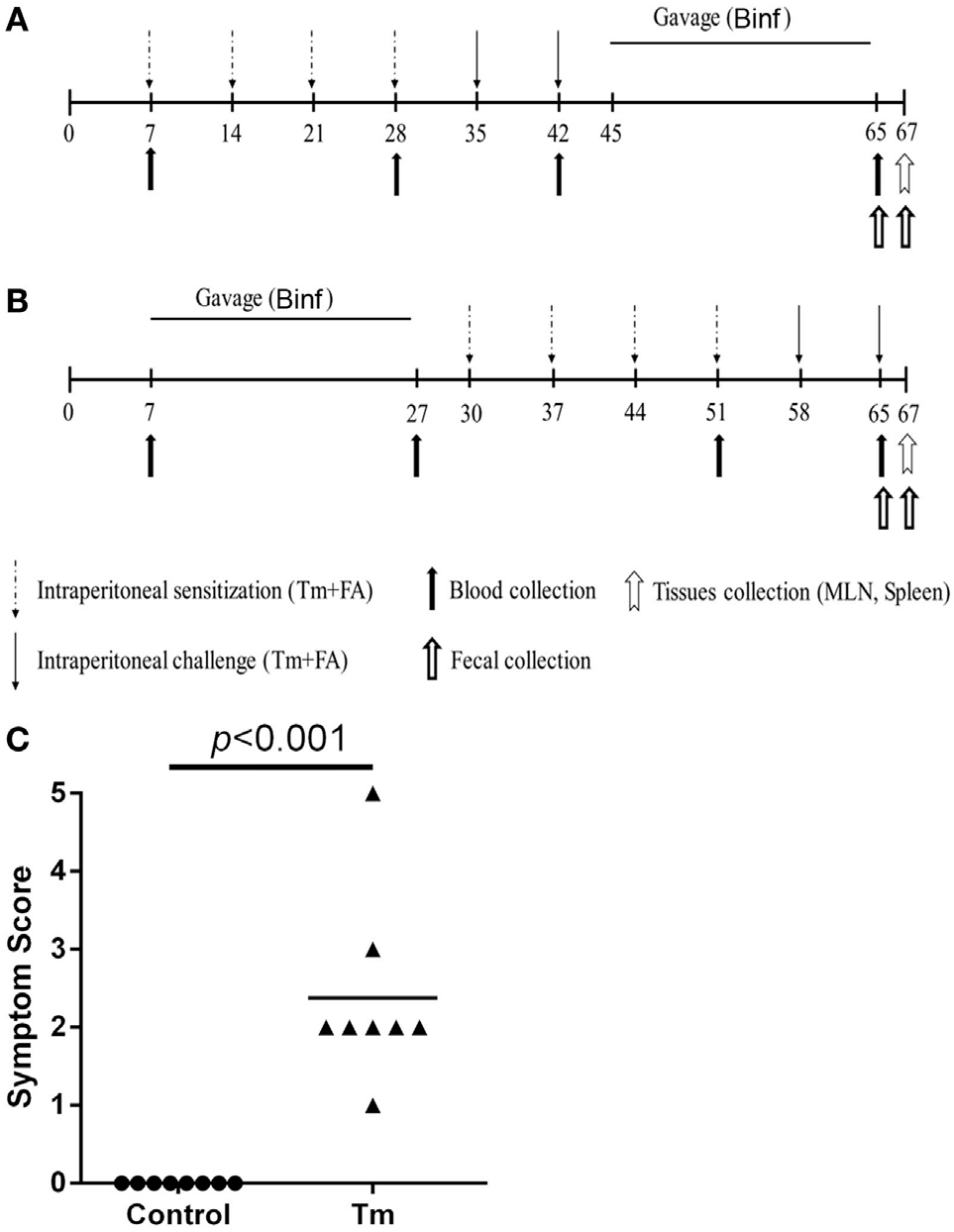
Immunization protocol of tropomyosin (Tm) sensitization model and gavage of Binf in BALB/c mice. Groups of 6- to 8-week-old female mice (*n* = 10) were sensitized four times by intraperitoneal injection with Tm (100 µg/mouse) plus Freund’s adjuvant (FA) and challenged twice with Tm (600 µg/mouse). **(A)** Therapeutic treatment group. Mice were sensitized on days 7, 14, 21, and 28 and challenged on days 35 and 42. Binf in phosphate-buffered saline (PBS) (10^9^ CFU/mouse) was administrated by gavage from days 45 to 65. Blood samples were taken from retro-orbital plexus on days 7, 28, 42, and 65. Fresh fecal samples were collected on days 45 and 65. **(B)** Preventive treatment group. Mice were sensitized on days 30, 37, 44, and 51 and challenged on days 58 and 65. Binf in PBS (10^9^ CFU/mouse) was administrated by gavage from days 7 to 27. Blood samples were taken from retro-orbital plexus on days 7, 27, 51, and 65. Fresh fecal samples were collected on days 30 and 65. On day 67, mice of all groups were sacrificed and tissues collected for immunological analysis. **(C)** The efficiency of the immunization model was assessed by symptom score.

For therapeutic treatment group, mice (*n* = 8) were intraperitoneally immunized with PBS containing purified Tm (100 µg/mouse) together with equal volume of complete Freund’s adjuvant (CFA) (Sigma, St. Louis, MO, USA) on days 7 and with Tm (100 µg/mouse) together with equal volume of incomplete Freund’s adjuvant (IFA) (Sigma, St. Louis, MO, USA) on days 14, 21, and 28, and challenged twice with Tm (600 µg/mouse) and equal volume of IFA on days 35 and 42. From days 45 to 65, mice were daily administered with 500 µl/mouse Binf (2 × 10^9^ CFU/ml) in PBS by gavage. For preventive treatment group, mice were daily administered with 500 µl/mouse Binf (2 × 10^9^ CFU/ml) from days 7 to 27, sensitized on day 30 with Tm (100 µg/mouse) together with equal volume of CFA, and on days 37, 44, and 51 with Tm (100 µg/mouse) together with equal volume of IFA, followed by challenging with Tm (600 µg/mouse) and equal volume of IFA on days 58 and 65. In either therapeutic group or preventive group, a corresponding positive control (*n* = 8) that given aseptic PBS instead of Binf was performed. In addition, a shared negative control (*n* = 8) for both therapeutic group and preventive group that given intraperitoneally injection of aseptic PBS (together with Freund’s adjuvant) instead of Tm by following the preventive protocol was performed and regarded as the unimmunized group. On day 67, mice were sacrificed for immunological analysis. The efficiency of the model was assessed by allergy symptom score (30). The protocol was illustrated in Figure 1.

### Serum Ig and Histamine Analysis

The blood were taken from retro-orbital plexus on days 7, 28, 42, and 65 for therapeutic treatment group and days 7, 27, 51, and 65 for preventive treatment group, centrifuged at 3,500 *g* for 10 min at 4°C, and the serum was collected and frozen at −80°C. Levels of serum Tm-specific IgE, IgG2a, and IgG1 were measured by ELISA as previously described with some modifications (31). In brief, serum samples were 1/20 diluted, and the secondary antibodies used in the ELISA tests were HRP-conjugated rat anti-mouse IgE, IgG2a, or IgG1 (Southern Biotechnology Associates, Birmingham, AL, USA). All the secondary antibodies were 1/6,000 diluted. After the HRP substrate TMB (eBioscience, San Diego, CA, USA) added, the absorbance was determined at 450 nm. Results were expressed as optical densities at 490 nm (OD490). Serum histamine was measured in 1/100 diluted serum samples with a commercial kit (Baomanbio, Shanghai, China) according to the manufacturer’s instructions, with a detection limit of 0.1 ng/ml.

### Bacterial DNA Isolation from Mouse Feces

Fresh fecal samples were collected immediately upon defecation under sterile condition in a 2-ml tube and stored at −80°C until processing for DNA isolation. Samples collected just before sacrifice (days 65 and 67 as duplication) were used for microbial analyses. Fecal samples from different individuals of the same group were combined (*n* = 8), then the DNA was isolated using a QIAamp Fast DNA Stool Mini kit (QIAgen, Valencia, CA, USA) according to the manufacturer’s instructions and then stored at −80°C until further use. DNA concentration was determined using a Nanodrop ND-1000 spectrophotometer (Thermo Fisher Scientific, San Jose, CA, USA), and the A260/A280 ratio between 1.8 and 2.0 was considered as a criterion for quality control.

### Metagenomic Sequencing and Bioinformatic Analyses

Isolated stool DNA was handled as previously reported (32) and sequenced by Sangon Biotech (Sangon Biotech, Shanghai, China). Briefly, amplification of the genomic DNA was performed using barcoded primers, which targeted the V3 to V4 region of the bacterial 16S rRNA gene. Fast length adjustment of short reads was used to merge overlapping paired-end Miseq fastq files (33). Sequences with mismatches in the overlapping region were discarded. The output fastq file was then analyzed by software PRINSEQ (34), and then chimeric reads were filtered using chimera.uchime (35). Reads were clustered into operational taxonomic units (OTU) using Uclust (36) at 97% pairwise identity threshold. Taxonomies were assigned to the representative sequence of each OTU using RDP classifier (37).

### Preparation of Lymphocytes

Spleens, Peyer’s patches (PPs) and MLNs were collected on sacrifice under sterile conditions. Single cell suspensions were prepared from spleen, PP, and MLN by pressing a piston and through a cell strainer, and the collected cells were washed with PBS. To isolate splenocytes, red blood cells were removed by RBC lysis buffer (Beyotime, Jiangsu, China). Then the lymphocytes and splenocytes were used as starting material for further analyses including RT-qPCR, ELISA, and flow cytometry.

### Flow Cytometry

Following *in vivo* treatments, single cell suspensions of spleen and MLN were washed and suspended in sterile PBS, and probed with anti-CD4-FITC, anti-CD25-PE, and anti-CD127-APC antibodies for Treg analysis, or with anti-CD69-FITC and anti-ST2-PE antibodies for Th2 analysis for 30 min at 4°C. For DC maturation analysis, mononuclear cells of spleen, PP and MLN were stained with anti-CD11c-FITC, anti-CD80-APC, anti-CD86-PE-Cy7, anti-CD103-PE, and anti-MHC-II-PerCP-eFlour 710 antibodies. All FACS reagents were purchased from eBioscience and used by following the manufacture’s recommendation. Preparations were acquired in flow cytometer (Gallios, Beckman Coulter, Fullerton, CA, USA) and results were analyzed with Flow Jo VX software (Tree Star Inc, Ashland, OR, USA).

### Isolation of CD11c^+^ DC and Naïve CD4^+^ T Cell

Six- to-eight-week-old female BALB/c mice were given Binf by intragastric administration with a dose of 10^9^ CFU/mouse for 20 days consecutively. The control mice were daily administered with equal volume of PBS. The positive control group mice were immunized four times and challenged twice as previously described. All the mice were killed by cervical dislocation. Spleen and MLN cell suspension were obtained as previously described. CD4^+^ cells were enriched from splenocytes with Mouse CD4^+^ T cell Isolation Kit (StemCell Technologies, Vancouver, BC, Canada), the enriched CD4^+^ fraction was subjected to cell sorting with FACSAria™ cell sorter (BD Bioscience, San Jose, CA, USA) to isolate CD4^+^CD62L^high^CD44^low^ naïve T cells, and the CD11c^+^ DC was isolated from cell suspensions of MLN in the same way (mouse DC Isolation Kit followed by cell sorting). Flow cytometry showed more than 99% purity of all the isolated cells. The antibodies used were anti-CD4-PE-Cyanine7, anti-CD62L-FITC, anti-CD44-APC and anti-CD11c-FITC (all from eBioscience, San Diego, CA, USA).

### *In Vitro* Co-Cultures

CD11c^+^ DCs (10^5^ cells/ml) isolated from MLNs of mice in each groups were cultured for 72 h with CD4^+^CD62L^high^CD44^low^ naïve T cells (10^6^ cells/ml) isolating from spleens of Tm-treated mice, in the presence of Tm (20 µg/ml) in RPMI 1640 culture medium (Gibco, Invitrogen, Grand Island, NY, USA) supplemented with 10% heat inactivated FBS (Tianhang, Hangzhou, China), 100 U/ml penicillin, 100 µg/ml streptomycin. T cells were polarized to Th1, Th2, Th17, and Treg subtypes *in vitro* by the addition of the following for 3 days: Th1, IL-12 (10 ng/ml; Peprotech, Rocky Hill, NJ, USA) and anti-IL-4 (10 µg/ml; eBioscience); Th2, IL-4 (10 ng/ml; Peprotech) and anti-IL-12 (10 µg/ml; eBioscience); Th17, TGF-β1 (0.2 ng/ml; Peprotech), IL-6 (40 ng/ml; Peprotech), anti-IFN-γ and anti-IL-4 (10 µg/ml; eBioscience); and Treg, TGF-β1 (0.2 ng/ml; Peprotech) and IL-2 (10 ng/ml; Peprotech). For APC-free T cell differentiation, plates were coated with anti-CD3 and anti-CD28 (10 µg/ml; eBioscience) at 4°C overnight, and then T cell with/without Binf (10^5^ CFU/ml) were differentiated to Th1, Th2, Th17, and Treg subtypes for 3 days with the same stimulators as in the DC-mediated polarization. After cultivation, precipitated cells were lysed and further used for RT-qPCR and ELISA analysis.

### Cytokine Assays

Cytokines (IFN-γ, IL-2, IL-4, IL-13, IL-17, IL-6, IL-10, and TGF-β) in the culture supernatants were determined by commercial ELISA kits (eBioscience) following the manufacturer’s recommendations.

### RT-qPCR

Total RNA was obtained using TRIzol reagent (Invitrogen, Life Technologies, Grand Island, NY, USA) and cDNA was synthesized with PrimeScript™ II 1st strand cDNA Synthesis kit (TaKaRa Shuzo, Ghiga, Japan). mRNA expression of each gene was measured with Light Cycler 480 SYBR Green I Master (Roche Applied Science, Indianapolis, IN, USA) using iTaq SYBR Green Supermix (TaKaRa). The housekeeping gene HRPT was included for normalization. The primer sequences were listed in Table S1 in Supplementary Material.

### Statistical Analysis

The Student’s *t*-test or ANOVA followed by Tukey’s *post hoc* test were used when appropriate for assessing the distribution of data. All results were expressed as the mean ± S.D. The SPSS 18.0 statistical software package (SPSS Inc., Chicago, IL, USA) was used for data analysis. *P* value of less than 0.05 was considered as significance.

To dig out the most relevant bacterial genera in Binf treatment, each of the five experimental groups (control, Tm [therapeutic], Tm + Binf [therapeutic], Tm [preventive] and Tm + Binf [preventive]) were positioned on the coordinate system, with specific bacterial genus proportion acting as *Y* value (the genera with a proportion of 0 were deleted) and Th2/Treg ratio acting as *X* value, then regular linear regression analysis was performed and the coefficient of correlation (*R*^2^) was calculated. Based on the *R*^2^, all the bacterial genera were arranged, and genera with the biggest *R*^2^ were considered as the most relevant ones (Table S2 in Supplementary Material).

## Results

### Binf Suppresses Tm-Induced Allergic Responses

**Figure 2:**
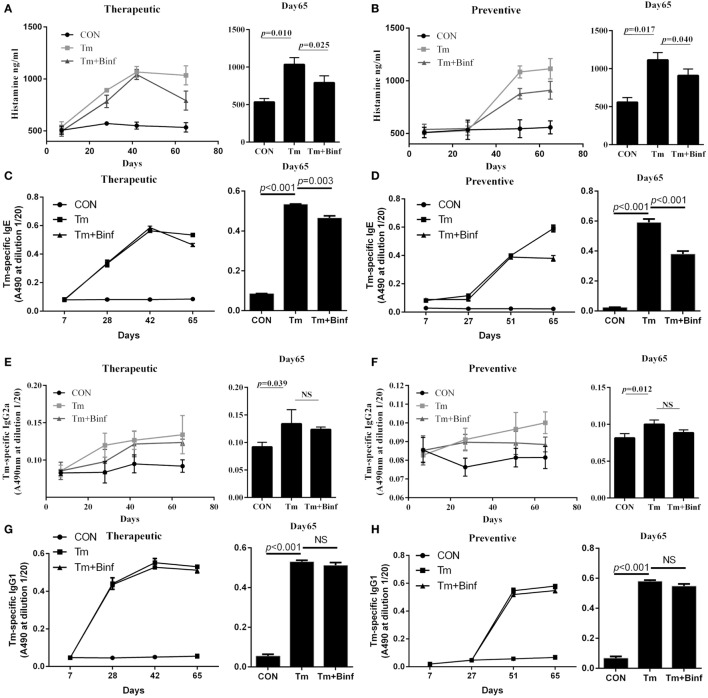
Effect of Binf intervention on the production of serum histamine and tropomyosin (Tm)-specific immunoglobulin *in vivo*. Serum were taken on days 7, 28, 42, and 65 for therapeutic treatment group and on days 7, 27, 51, and 65 for preventive treatment group (*n* = 8). Histamine **(A,B)**, Tm-specific IgE **(C,D)**, Tm-specific IgG2a **(E,F)**, and Tm-specific IgG1 **(G,H)** were measured. *P* values determined by one-way ANOVA followed by Tukey’s *post hoc* test. CON, control group; Tm, Tm-treated group; Tm + Binf, Binf intervened group; NS, not significant.

In the initial study, we evaluated the effect of probiotic Binf in modulating Tm-induced allergic responses in a mouse model (Figure 1). The treatment was divided into two groups: the therapeutic group that applied Binf after Tm stimulation (Figure 1A) and the preventive group that applied Binf before Tm stimulation (Figure 1B). Tm-treated mice showed strong allergic symptom, indicating the validity of the model (Figure 1C). Compared to the control, Tm-sensitized mice produced high levels of serum histamine, Tm-specific IgE, IgG2a, and IgG1 over the time-course of the experiment (Figure 2). At the end of entire experimental period (day 65), the levels of histamine (*p* < 0.05) and Tm-specific IgE (*p* < 0.001) were significantly lower in mice supplemented with oral administration of Binf in both therapeutic and preventive ways compared with Tm stimulation group (Figures 2A–D), while no significant difference found in the level of Tm-specific IgG2a and IgG1 between Tm + Binf and Tm groups (Figures 2E–H). Since the production of histamine and specific IgE are typical allergic responses, these results indicated that oral administration of Binf suppresses Tm-induced allergy in both preventive and therapeutic ways.

### Binf Increases Tregs for Balancing Th2/Treg

**Figure 3:**
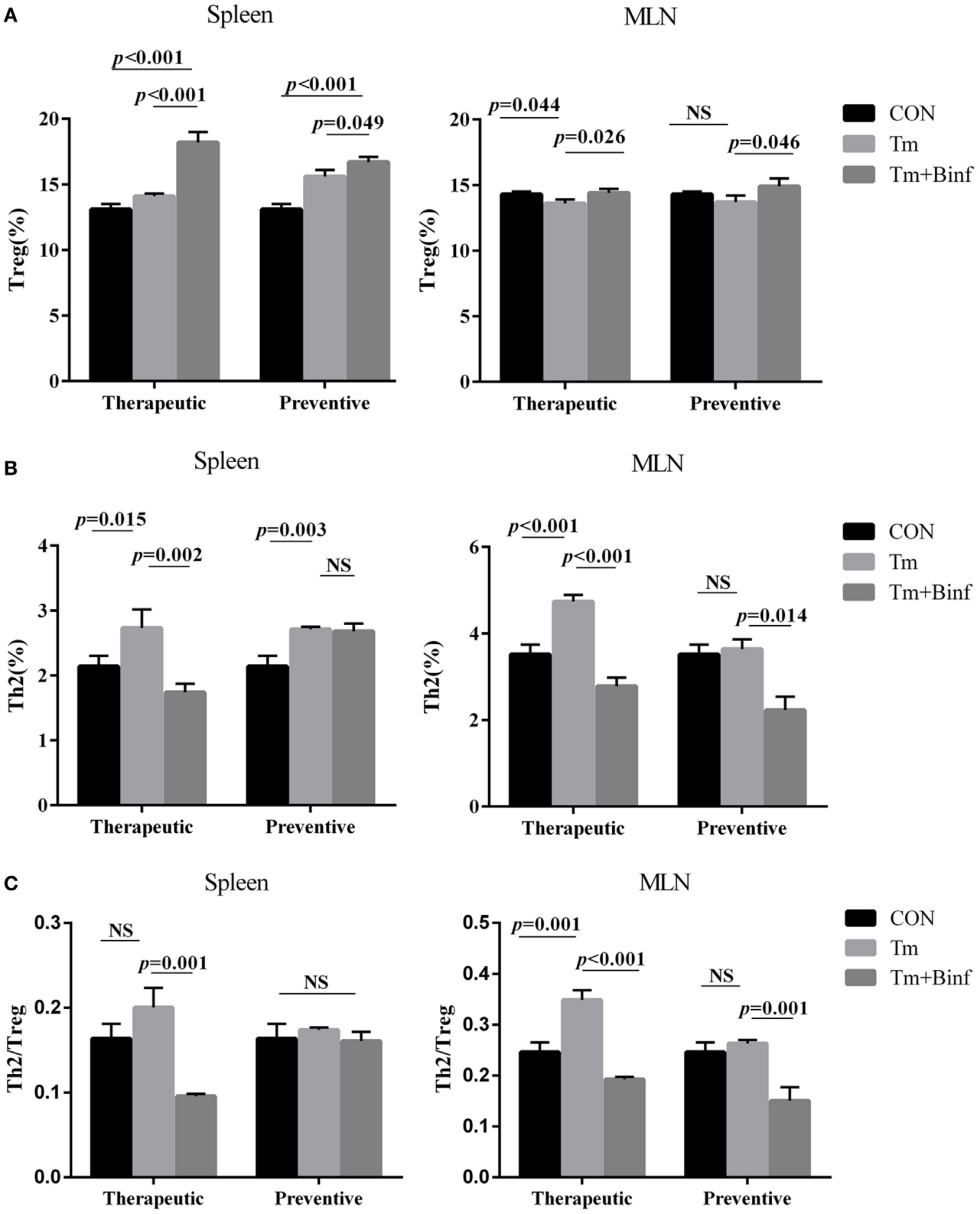
Binf modulates Th2 and regulatory T cell (Treg) proportion in mesenteric lymph node (MLN) and spleen. Lymphocytes isolated from MLN and spleen from each group were analyzed by flow cytometry. The percentages of CD4^+^CD25^+^CD127^low/−^ Treg **(A)** and CD4^+^CD69^+^ST2^+^ Th2 **(B)** in the whole CD4^+^ T cell population from MLN or spleen were measured (*n* = 5), and ratios of the percentages of Th2 to Treg were calculated **(C)**. *P* values determined by one-way ANOVA followed by Tukey’s *post hoc* test. CON, control group; Tm, Tm-treated group; Tm + Binf, Binf intervened group; NS, not significant.

To test whether Binf can induce Treg cells and affect Th2/Treg balance *in vitro*, we investigated the proportion of Th2 (CD4^+^CD69^+^ST2^+^) and Treg (CD4^+^CD25^+^CD127^low/−^) cells in spleen and MLN whole CD4^+^ T cell population by flow cytometry. As shown in Figure 3 and Figure S2 in Supplementary Material, in both therapeutic and preventive groups, administration of Binf significantly increased the proportion of Treg in spleen and MLN upon Tm challenge (Figure 3A). By contrast, the proportion of Th2 significantly decreased after Binf treatment (Figure 3B).

Furthermore, to eliminate the interference of total CD4^+^ T cells proliferation, we calculated the ratio of Th2/Treg, which can counteract the variation of the whole CD4^+^ T cell population and emphasize the relevant abundance of allergic Th2 and anti-allergic Treg. The results showed that in most cases, Tm challenge increased Th2/Treg ratio, and Binf treatment significantly decreased that ratio and maintained Th2/Treg balance (Figure 3C). However, no significant changes of Th2/Treg ratio in spleen of the preventive group were observed, indicating the less important role of Th2 and Treg in the situation.

Overall, these results demonstrated that Binf promotes the induction of Tregs and balance Th2/Treg for suppressing Th2 responses in Tm-sensitized mice.

### Binf Promotes DCs Maturation and Tolerogenic DCs Accumulation

**Figure 4:**
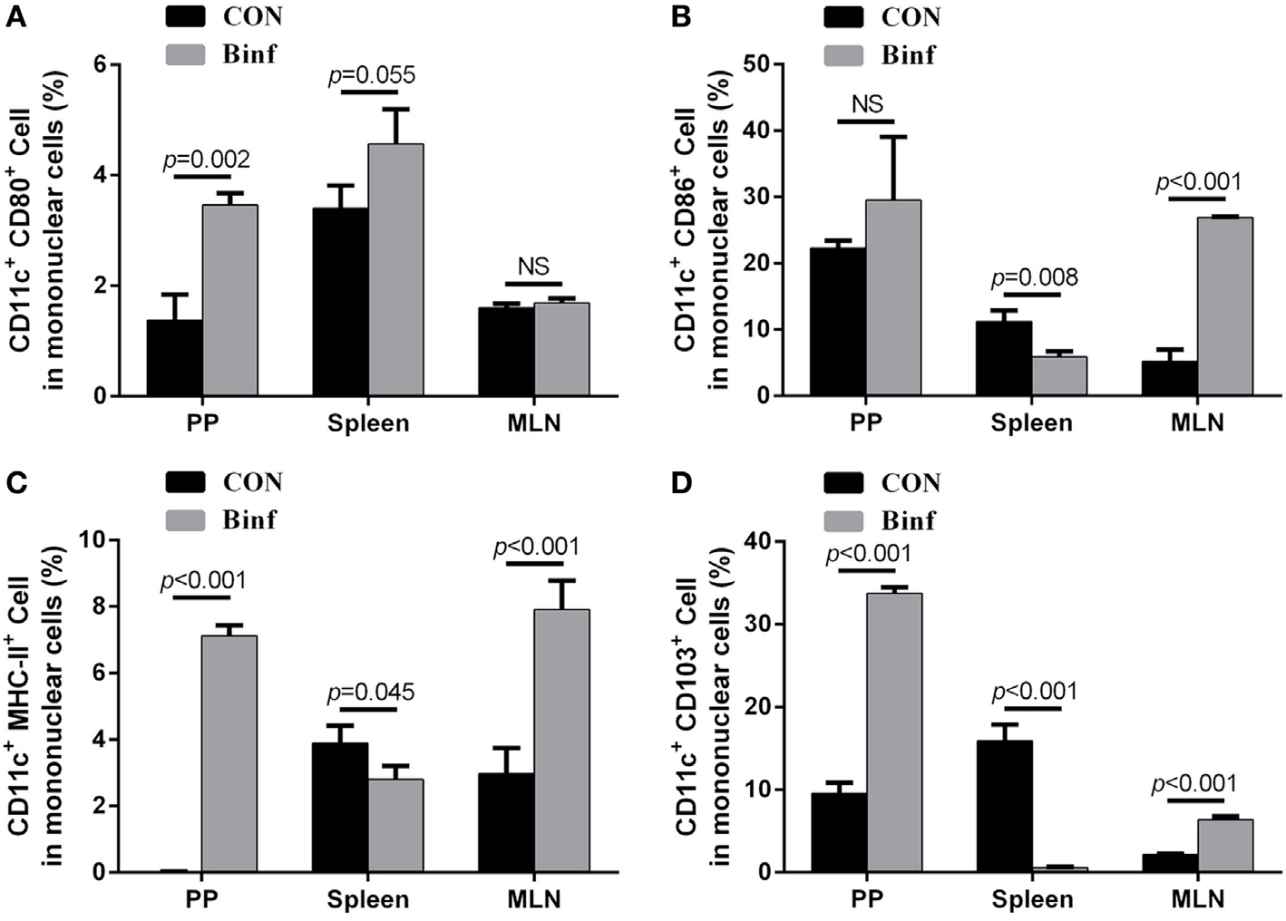
Binf promotes dendritic cell (DC) maturation and induces CD103^+^ DC accumulation. BALB/c mice were given Binf by intragastric administration with a dose of 10^9^ CFU/mouse for 20 days consecutively. The control mice were given phosphate-buffered saline instead. Lymphocytes isolated from Peyer’s patch (PP), mesenteric lymph node (MLN), and spleen from both groups were determined by flow cytometric analysis. The percentage of CD11c^+^ and CD80^+^ **(A)**, CD86^+^ **(B)**, MHC-II^+^ **(C)**, or CD103^+^ **(D)** cells in mononuclear cells from PP, MLN, and spleen were shown. *P* values determined by Student’s *t*-test; NS, not significant.

Based on the expression of cell surface markers, abundant subsets of DCs have been described (38, 39). Interestingly, the functions of different DC subsets on the stimulation of T cells are tissue dependent (40–42). To explore the role of Binf in activating functional DCs, BALB/c mice were treated with probiotic Binf by intragastric administration at a dose of 10^9^ CFU/mouse for consecutive 20 days, and the percentage of CD80^+^, CD86^+^, MHC-II^+^, and CD103^+^ cells with CD11c^+^ DC marker in mononuclear cell population were measured by flow cytometry (Figure 4; Figure S3 in Supplementary Material).

The intake of Binf significantly increased the percentages of CD80^+^ (Figure 4A), CD86^+^ (Figure 4B), and MHC-II^+^ (Figure 4C) DCs in PP and MLN, indicating the role of Binf in maturation of DCs in GALT. However, the changes of different DC subsets in spleen by Binf treatment were inconsistent, suggesting the less efficient role of Binf in peripheral immune system. In addition, Binf largely increased CD103^+^ DCs population in PP and MLN, while in contrast, decreased that in spleen (Figure 4D). On the basis of these observations, we concluded that Binf promotes DCs maturation and tolerogenic CD103^+^ DCs accumulation in GALT, which is involved in the regulation of Treg and Th cell differentiation, but Binf effect on spleen DCs is mild and needs far more investigations.

### Binf Stimulates DCs on T Cell Differentiation *In Vitro*

**Figure 5:**
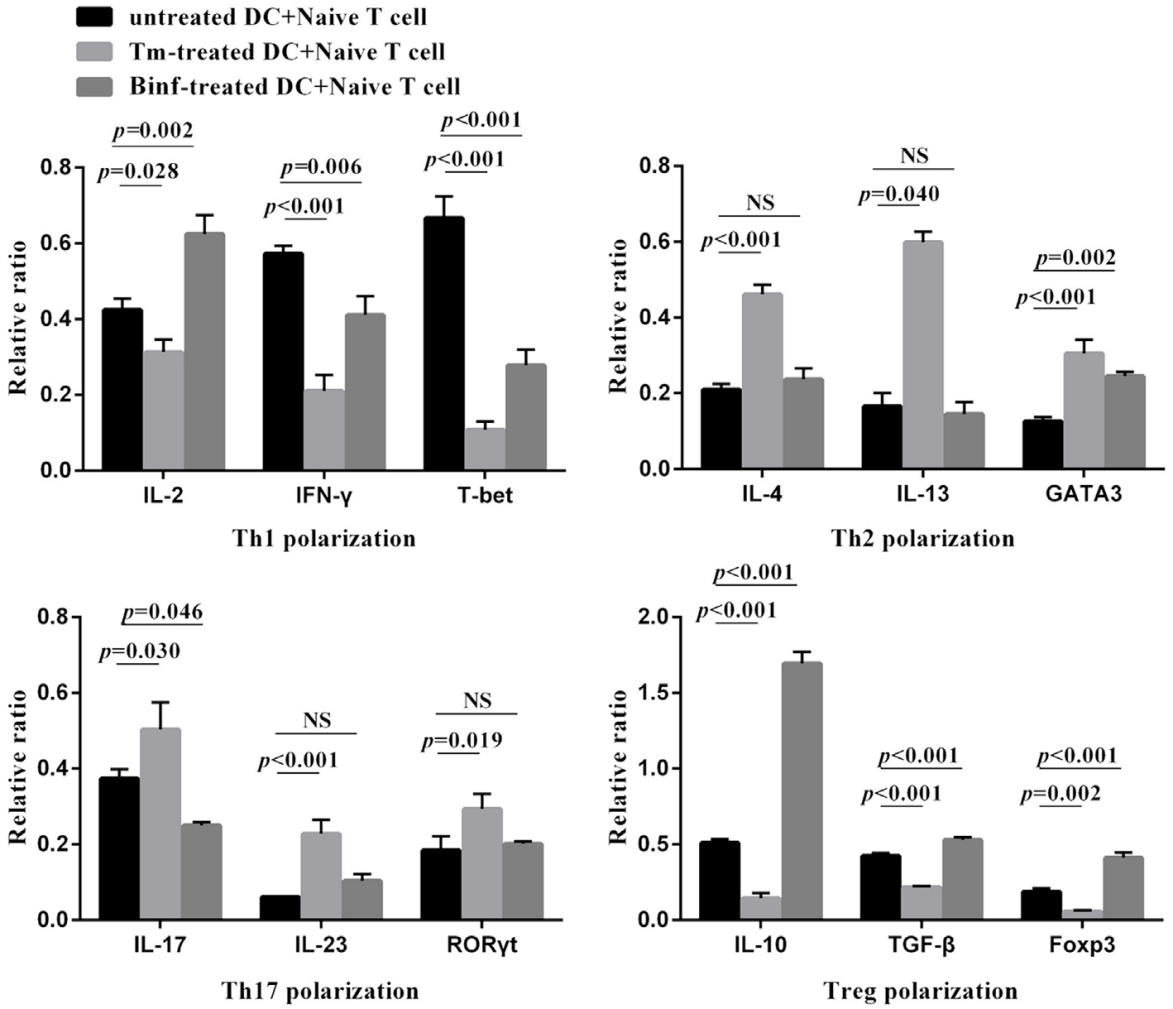
Binf stimulates dendritic cells (DCs) on T cell differentiation *in vitro*. CD4^+^ CD62L^high^ CD44^low^ naïve T cells were co-cultured with CD11c^+^ DCs isolated from phosphate-buffered saline (PBS)-, tropomyosin (Tm)-, or Binf-treated mouse in Th1, Th2, Th17, or Treg polarization culture conditions for 3 days. mRNA expression of corresponding cytokines (IFN-γ, IL-2, IL-4, IL-13, IL-17, IL-23, IL-10, and TGF-β) and transcription factors (T-bet, GATA3, RORγt, and Foxp3) were measured by RT-qPCR, the housekeeping gene HRPT was included for normalization. *P* values were calculated by one-way ANOVA followed by Tukey’s *post hoc* test. Data are representative of three independent experiments. NS, not significant.

To investigate the role of stimulated MLN DCs in the differentiation of naïve T cells into other CD4^+^ T-cell subsets, naïve CD4^+^ T cells were co-cultured with CD11c^+^ DCs isolated from MLNs of control, Tm-treated or Binf-treated mice in the presence of specific stimulators (IL-12 and anti-IL-4 antibody for Th1 polarization; IL-4 and anti-IL-12 antibody for Th2 polarization; TGF-β1, IL-6, anti-IFN-γ, and anti-IL-4 antibodies for Th17 polarization; TGF-β1 and IL-2 for Treg polarization). The mRNA levels of the typical cytokine and master regulator genes of different T cell subsets were measured by RT-qPCR (Figure 5).

Tropomyosin challenge significantly downregulated the mRNA expression of Th1- and Treg-typical marker genes, while upregulated the Th2- and Th17-typical ones, indicating inflammation and allergic responses were provoked. Notably, Binf significantly promoted the mRNA expression of IL-10, TGF-β, and Foxp3 under Treg-polarizing conditions, demonstrating that Binf preferentially induces Treg cell differentiation in the presence of mature DCs.

### DCs Are Required for Binf-Specific Induction of Tregs

**Figure 6:**
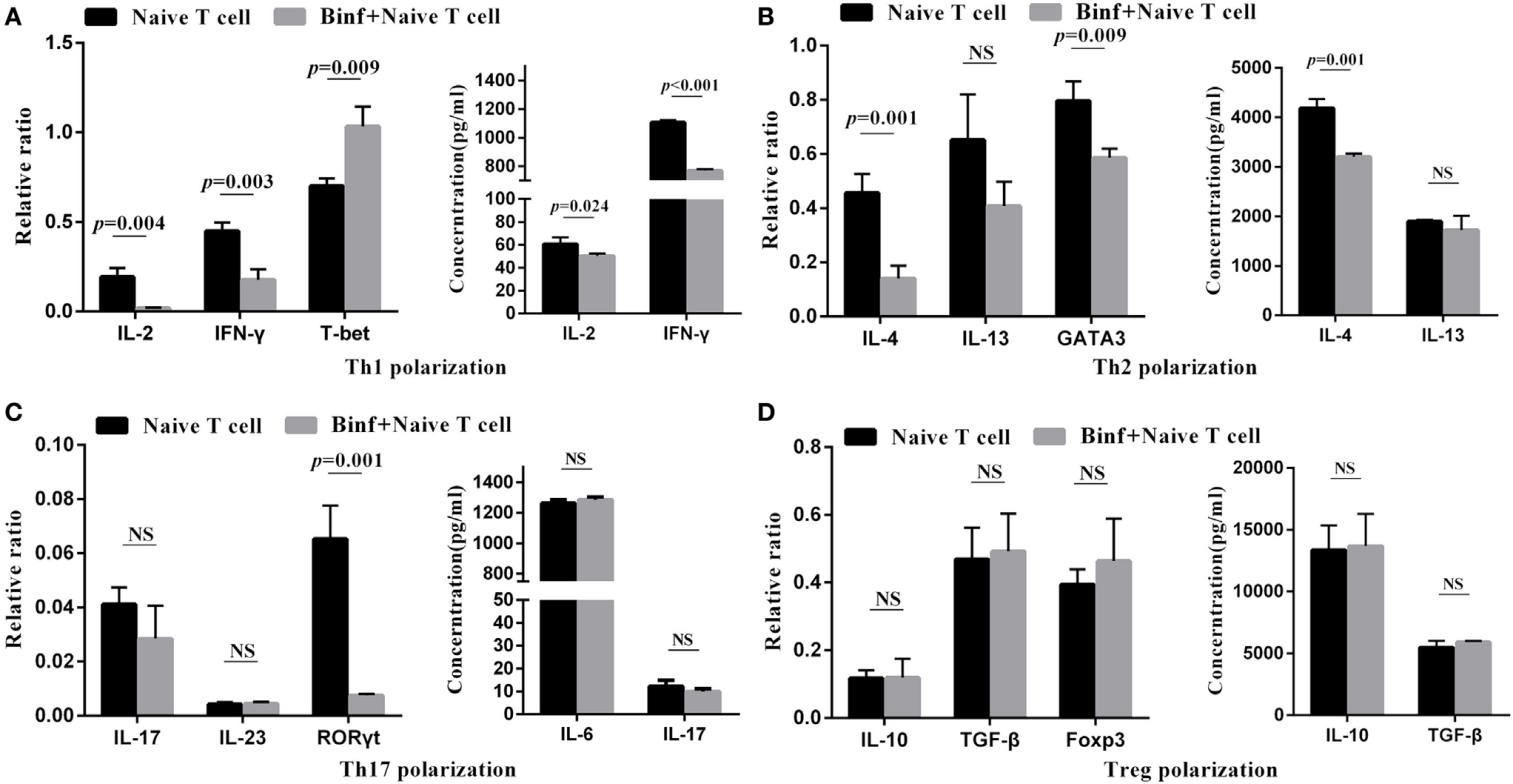
Direct effect of Binf on T cell differentiation *in vitro*. CD4^+^ CD62L^high^ CD44^low^ naïve T cells were cultured with anti-CD3 and anti-CD28 antibodies, under the Th1, Th2, Th17, or regulatory T cell (Treg) skewing culture conditions, and in the presence or absence of Binf for 3 days. mRNA expression of Th1 **(A)**, Th2 **(B)**, Th17 **(C)**, and Treg **(D)** cytokines and transcription factors were measured by RT-qPCR (left panels, normalized by housekeeping gene HRPT) and ELISA (right panels). *P* values were calculated by one-way ANOVA followed by Tukey’s *post hoc* test. Data are representative of three independent experiments. NS, not significant.

To investigate the role of DCs in T cell differentiation by Binf, we replaced DCs with anti-CD3 and anti-CD28 antibodies to mimic DCs costimulation signals in the *in vitro* T cell differentiation assay. In the presence of Binf, the mRNA expression of T-bet (Th1 master regulator) significantly enhanced, while that of GATA3 and RORγt, master regulators of Th2 and Th17, respectively, significantly reduced (Figure 6). Interestingly, treatment with Binf largely lowered the Th1 cytokines (IL-2 and IFN-γ) at both transcriptional and translational levels (Figure 6A). In addition, Binf showed a moderate effect on the expression of Th2 cytokines due to a significant decrease of IL-4 but not IL-13 observed (Figure 6B), whereas no effect of Binf on Th17 cytokines (IL-6, IL-17, and IL-23) expression can be found (Figure 6C). Importantly, treatment with Binf did not have any effect on the expression of IL-10, TGF-β, and Foxp3 at both transcriptional and translational levels in the absence of DCs (Figure 6D), indicating that the presence of DCs is a prerequisite for Treg cell induction by Binf.

### Binf Modulates Fecal Microbiota Composition in Tm-Sensitized Mice

We further performed metagenomic analysis of fecal microbiota composition with or without Binf treatment under Tm challenge to explore the effect of Binf on alterations of gut microbiota, which in turn might affect the induction of Tregs.

**Figure 7:**
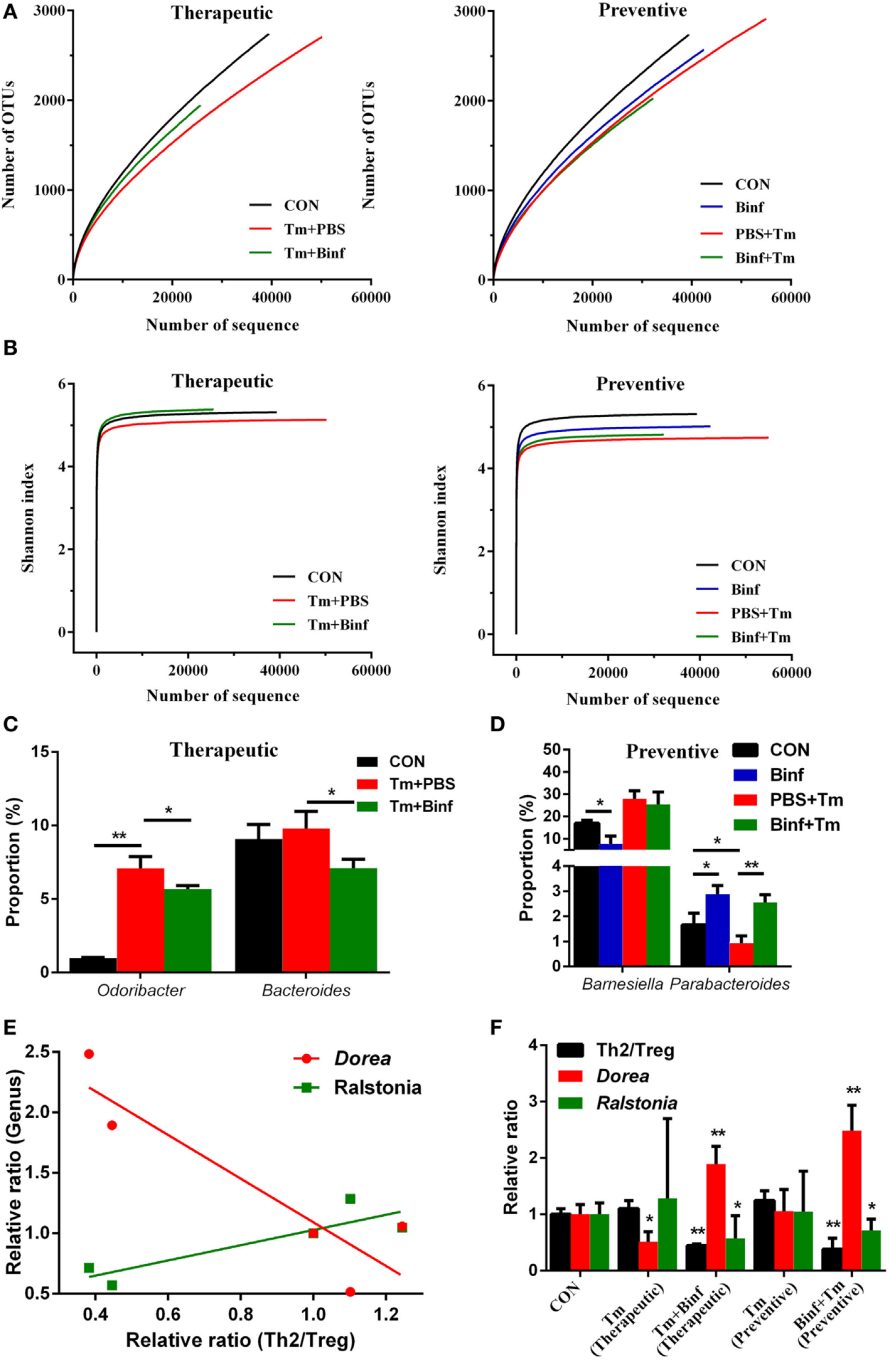
Binf modulates fecal microbiota composition in tropomyosin (Tm)-sensitized mice. Genomic DNA was extracted from the fecal samples taken from control (CON), preventive, and therapeutic treatment groups just before sacrifice. **(A)** The microbiota richness of each group. **(B)** The microbiota diversity of each group. **(C)** Significantly changed microbial genera in the therapeutic group. **(D)** Significantly changed microbial genera in the preventive group. **(E)** Two of the most significant genera in the linear regression analysis of microbiota proportion and Th2/Treg ratio. **(F)** The relative ratio of Th2 to regulatory T cell (Treg), *Dorea*, and *Ralstonia* proportion in each sample groups, the significance between control and each treatment groups were calculated. *p < 0.05; **p < 0.01.

As shown in Figure 7A, in the therapeutic group, measurements of ecological metrics revealed that Tm diminished the richness of fecal microbiota in mice, but that could be partially restored by Binf administration. However, in the preventive group, Binf did not have such a significant effect on improving richness of commensal microbes. Unexpectedly, preventive administration of Binf without Tm challenge reduced the richness of fecal microbiota compared to the control, while that with Tm challenge (Binf + Tm) compromised the loss of richness caused by Tm sensitization (PBS + Tm) (Figure 7A). Consistently, the effect of Binf on the diversity of gut microbiota showed identically as that on the richness (Figure 7B).

Furthermore, we also analyzed gut microbiota at various taxonomic levels to investigate the role of Binf in the distribution change of predominant bacterial species. At the genera scale, Binf relieved Tm sensitization-caused proportion imbalance of *Odoribacter* and *Bacteroides* in the therapeutic group (Figure 7C; Figure S4A in Supplementary Material). In the preventive group, two genera that are designated as *Barnesiella* and *Parabacteroides* can be regulated by the intake of Binf (Figure 7D; Figure S4A in Supplementary Material). Specifically, Binf significantly decreased *Barnesiella* proportion directly, but the effect under Tm treatment is slight. By contrast, the proportion of *Parabacteroides* decreased by Tm sensitization and can be restored by Binf. At the phyla level, we found that the therapeutic intake of Binf could balance the ratio of *Bacteroidetes* vs. *Firmicutes* disordered by Tm challenge. However, direct intake of Binf will decrease the *Firmicutes*/*Bacteroidetes* ratio and thus deteriorate the imbalance induced by Tm challenge in preventive administration (Figures S4B,C in Supplementary Material). These data demonstrated the importance of gut microbiota in mediating the alleviation of food allergy by Binf.

In addition to assess the effect of Tm and Binf on the predominant microbiota bacterial species, we also tried to reveal the genera that most significantly correlated with Tm-induced allergy and Binf intervention. By using linear regression analysis, we found *Dorea* and *Ralstonia* were tightly correlated with the lymphocytes pattern (Th2/Treg ratio) (Figures 7E,F; Table S2 in Supplementary Material), which had the coefficient of determination (*R*^2^) of 0.82 and 0.77, respectively. Interestingly, *Dorea* and *Ralstonia* showed the opposite correlation with the lymphocytes pattern: Th2/Treg ratio was negatively correlated with *Dorea*, but positively correlated with *Ralstonia*, when compared within each group in both therapeutic and preventive ways. Therefore, we propose that Binf probably alleviates Tm-caused allergic response through the increase of *Dorea* and decrease of *Ralstonia*, which is involved in Treg cell differentiation and balancing Th2/Treg ratio. But more studies are needed to fully reveal the exact role of these bacteria in modulating allergy responses, the detailed relationship between *Dorea, Ralstonia*, and T cell differentiation, and the underlying mechanisms.

## Discussion

Despite probiotics administration has been shown to be an effective strategy for the prevention of allergic sensitization in experimental and clinical studies, little information is available on mechanisms of their action. Besides, there is a significant difference in effects with long-term supplementation of probiotics in relation to species and even the strain. Therefore, in the present work, we investigated the effect of Binf in alleviating Tm-induced allergy and clarified the possible mechanism underlying through *in vivo* and *in vitro* studies.

We first set up a mouse model of Tm-induced allergy. When treated with probiotic strain Binf, production of histamine and Tm-specific IgE but not IgG2a or IgG1 was reduced. Because IgE is Th2 associated and the major antibody in allergic reactions, while IgG2a and IgG1 is Th1 associated and does not participate in allergic stimulating, the inhibition of IgE but not IgG2a or IgG1 exactly exhibited the specific anti-allergy activity of Binf. In addition, we also found that Binf increased CD4^+^CD25^+^CD127^low/−^ Tregs proportion for balancing Th2/Treg ratio in this mouse model. CD4^+^CD25^+^CD127^low/−^ Tregs have been reported to be the “real” natural Tregs with the highest expression level of Foxp3 and the strongest inhibitory effect on responder T cells (43). Therefore, the effect of Binf in inhibiting allergic reactions in this study attributes to the induction of CD4^+^CD25^+^CD127^low/−^ Tregs with highly strong function of suppressing Th2-predominant and even other inflammatory responses.

Furthermore, by co-culture of CD11c^+^ DCs and naïve T cells, we found that Tm significantly increased Th2- and Th17-regulated response while decreased Th1- and Treg-regulated response, demonstrating the strong pro-allergic ability of Tm in this *in vitro* co-culture cell model. We also found that Binf mainly induce Tregs but not the other cell types. Alternatively, in the absence of DCs, the induction of Tregs by Binf was no longer observed, indicating that process is DC dependent. Correspondingly, in the mouse model, our results showed that Binf promotes DCs maturation and CD103^+^ DCs accumulation to GALT, and it have been reported that CD103^+^ DCs in GALT were able to mediate the conversion of naïve T cells into Tregs (44). Collectively, these data indicated that Binf promotes Tregs differentiation by inducing tolerogenic DCs. However, more studies are needed to elucidate the underlying mechanism of the maturation and accumulation of DCs induced by Binf.

Moreover, in the presence of Binf but absence of DCs, the levels of master regulator genes of Th1 (T-bet), Th2 (GATA3), and Th17 (RORγt) significantly changed (T-bet was upregulated while GATA3 and RORγt were downregulated); however, the gene levels of T-cell response-associated effectors (cytokines) did not change consistently. It is known that these master regulator genes are responsible for functional T cell subsets differentiating from naïve T cell, and the cytokines were produced by differentiated T cells (45). Therefore, we propose that Binf could directly regulate the differentiation of Th1, Th2, and Th17 cells, but do not exclude the possibility that the subsequent T cell functions might be controlled by other factors and not fully rely on Binf stimulation.

Published data have demonstrated that gut microbiota is tightly correlated with allergic diseases (17, 46). Consistently, we also found that Binf can modulate gut microbiota composition, which may further affect immune responses in Tm sensitization. We found that different bacteria genera or phyla showed different patterns under Tm challenge or Binf administration, suggesting the different roles of those bacteria in allergic and immune responses. Particularly, genus *Dorea* was tightly negatively correlated with Th2/Treg ratio, assuming *Dorea* may contribute to induction of Tregs and suppress Tm-induced allergic reaction; in sharp contrast, genus *Ralstonia* was positively correlated with Th2/Treg ratio, and thus might inhibit Treg differentiation and promote Tm-induced allergy. Consistently, previous research has also highlighted the role of *Dorea* and *Ralstonia* in food allergy and immunity. A 3-year follow-up study showed that *Dorea* is reduced in the intestinal microbiomes of infants who later develop food sensitization or food allergy, and thus suggested *Dorea* may protect against food sensitization and food allergy (47). On the contrary, *Ralstonia* was considered to be proinflammatory during Parkinson’s disease (48), and *Ralstonia* was also relatively more abundant in asthmatic compared with non-asthmatic chronic rhinosinusitis patients (49), implying the activity of *Ralstonia* in disrupting host immune system. However, all these studies as well as our results only revealed the relationship between these bacteria and T cell population, and the underlying causal relationship needs far more researches to investigate. Notably, Binf supplementation did not increase Binf proportion but altered other bacterial composition of gut microbiota, indicating an indirect function of Binf in modulating the gut microbiota composition. Based on this observation, we conclude that Binf directly promote DCs and Tregs or indirectly modulate gut microbiota composition to extinguish allergic responses. Thus, intestinal DCs, Tregs, and bacteria genera *Dorea* might be useful to develop novel therapeutic strategy for Tm-induced allergy and even other kinds of hypersensitivities.

**Figure 8:**
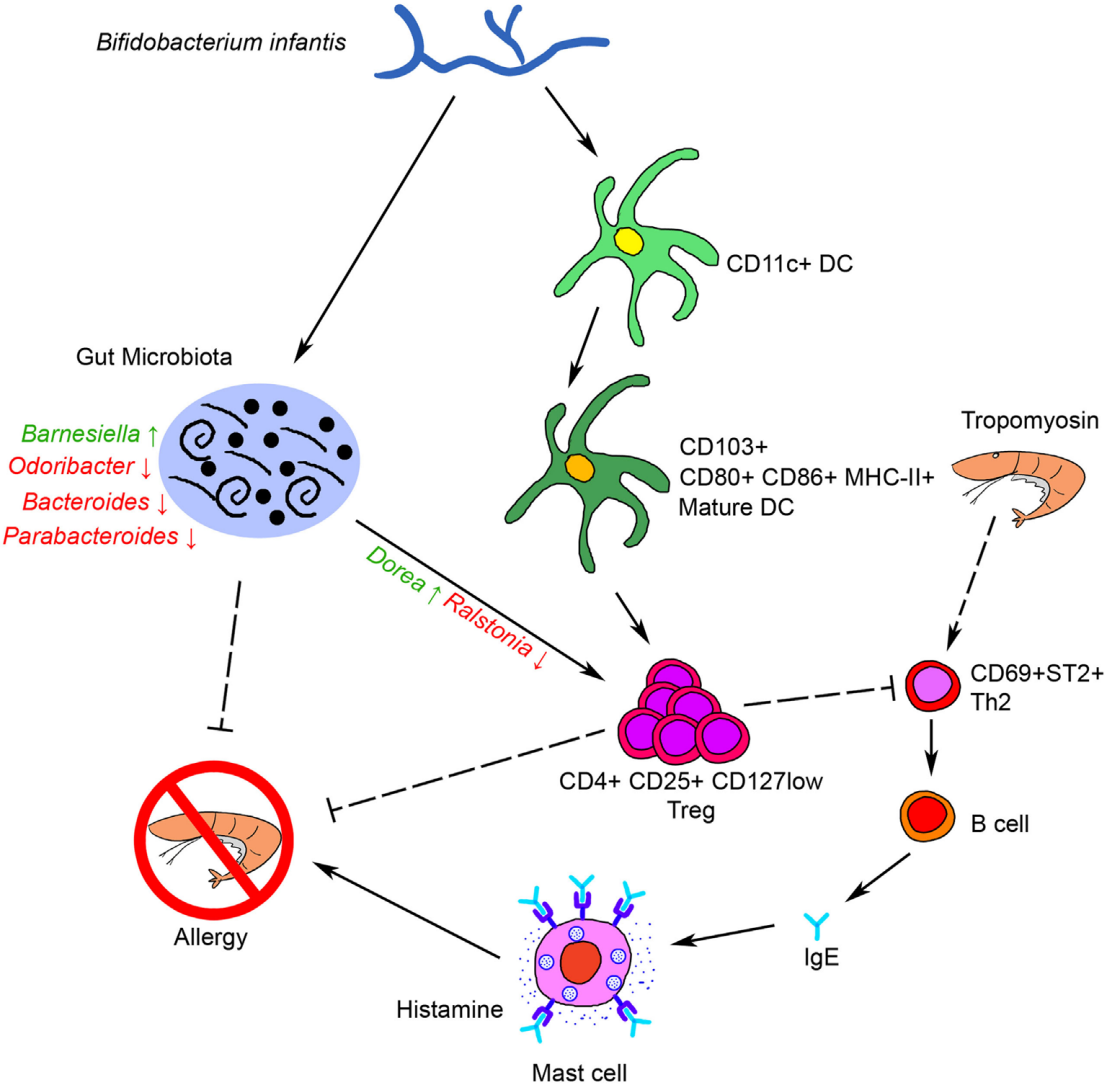
Schematic representation of the anti-allergy activity of Binf. Binf promotes dendritic cells (DCs) maturation and CD103^+^ tolerogenic DCs accumulation in gut-associated lymphoid tissue, and modifies the gut microbiota composition, especially upregulates *Dorea* proportion and downregulates *Ralstonia* proportion. Both of the two ways further induce regulatory T cell (Treg) polarization and finally inhibit tropomyosin-induced allergy.

Taken together, our results showed that oral administration of Binf suppresses Tm-induced allergic response in a mouse model through several possible ways as bellows: on the one hand, Binf promotes DCs maturation and tolerogenic CD103^+^ DCs accumulation in GALT, which further modulates T cell differentiation, especially induces Tregs for suppressing Th2 response; on the other hand, Binf regulates the alterations of gut microbiota composition, especially the increase of *Dorea* and decrease of *Ralstonia*, which is also involved in Th2/Treg balancing. Finally, immune tolerance was triggered and mucosal Th2 responses to food antigens and intestinal allergic reactivity were inhibited (Figure 8). Our findings not only link probiotic Binf to commensal microbiota population and the induction of functional Treg cells in the mucosa, but also provide molecular insight into the application of Binf in alleviating food allergy and even gut immune homeostasis.

## Ethics Statement

This study was carried out in strict accordance with the recommendations in the National Guide for the Care and Use of Laboratory Animals of China. All animal procedures were approved by the Zhejiang Gongshang University Laboratory Animal Welfare Ethics Review Committee.

## Author Contributions

LF, JS, and YW conceptualized the study; LF and YW drafted the work and revised it critically for important intellectual content; LF, JS, CW, and SF acquired and analyzed the data and drafted the manuscript; LF and YW revised the manuscript critically and provided overall supervision.

## Conflict of Interest Statement

The authors declare that the research was conducted in the absence of any commercial or financial relationships that could be construed as a potential conflict of interest.

## Supplementary Material

Figure S1 Shrimp tropomyosin (Tm) purification from *Litopenaeus vannamei*. Protein products from each purification step were detected by SDS-PAGE and coomassie blue staining. Lanes 1–4, the supernatant of each extracting procedure by Buffer A; lane 5, the supernatant of extracting procedure by Buffer B; lane 6, the supernatant after heat treatment; lane 7, the final Tm product.

Figure S2 Binf modulates Th2 and regulatory T cell (Treg) proportion in mesenteric lymph node (MLN) and spleen. **(A)** Flow cytometry results for Treg (CD4^+^CD25^+^CD127^low/−^) in spleen and MLN. **(B)** Flow cytometry results for Th2 (CD69^+^ST2^+^) in spleen and MLN.

Figure S3 Binf promotes dendritic cell (DC) maturation and induces CD103^+^ DC. CD80^+^, CD86^+^, MHC-II^+^, and CD103^+^ DCs (CD11c^+^) proportion in each groups was measured by flow cytometry.

Figure S4 Effect of Binf on fecal microbiota composition in tropomyosin (Tm)-sensitized mice. **(A)** Sequencing analysis of fecal microbiota composition from mice at genera level. **(B)** Sequencing analysis of fecal microbiota composition from mice at phyla level. **(C)** The ratio of two most abundant phyla (*Firmicutes*/*Bacteroidetes*) in each group.

## Abbreviations

Tm, shrimp tropomyosin; Binf, *Bifidobacterium infantis* 14.518; GALT, gut-associated lymphoid tissue; Th, T helper cell; Treg, regulatory T cell; DC, dendritic cell; FA, Freund’s adjuvant; OD, optical density; OTU, operational taxonomic units; PP, Peyer’s patch; MLN, mesenteric lymph node.
