# Supplementary material for: Bifidobacterium infantis Potentially Alleviates Shrimp Tropomyosin-Induced Allergy by Tolerogenic Dendritic Cell-Dependent Induction of Regulatory T Cells and Alterations in Gut Microbiota
Source: Front Immunol. 2017 Nov 10;8:1536. doi: 10.3389/fimmu.2017.01536 (PMC5686061; doi:10.3389/fimmu.2017.01536)
Supplement: Supplementary file 1 [file table_1.docx]

**Table S1.** Primer sequences used in RT-qPCR.

| **Genes** | **Forward primer (5’ – 3’)** | **Reverse primer (5’ – 3’)** |
| --- | --- | --- |
| Foxp3 | AGAGTTCTTCCACAACATGGACTACTT | GATGGCCCATGGATAAGG |
| GATA-3 | CTTATCAAGCCCAAGCGAAG | CCCATTAGCGTTCCTCCTC |
| HRPT | CTGGTGAAAAGGACCTCTCG | TGAAGTACTCATTATAGTCAAGGGCA |
| IFN-γ | TGGCATAGATGTGGAAGAAAAGAG | TGCAGGATTTTCATGTCACCA |
| IL-10 | GGTTGCCAAGCCTTATCGGA | ACCTGCTCCACTGCCTTGCT |
| IL-13 | AGACCAGACTCCCCTGTGCA | TGGGTCCTGTAGATGGCATTG |
| IL-17A | AGGGAGAGCTTCATCTGTGG | AGATTCATGGACCCCAACAG |
| IL-2 | CACATTTGAGTGCCAATTCGAT | GCGCTTACTTTGTGCTGTCCTA |
| IL-23 | TGCTGGATTGCAGAGCAGTAA | GCATGCAGAGATTCCGAGAGA |
| IL-4 | ACAGGAGAAGGGACGCCAT | GAAGCCCTACAGACGAGCTCA |
| RORγt | AGAAAGAAAAGGGGAACTGG | CTATTGTGGCTGCTGAGTTC |
| T-bet | TCAACCAGCACCAGACAGAG | AACATCCTGTAATGGCTTGTG |
| TGF-β | ACCGCAACAACGCCATCTAT | GCACTGCTTCCCGAATGTCT |
