# Supplementary material for: Bifidobacterium infantis Potentially Alleviates Shrimp Tropomyosin-Induced Allergy by Tolerogenic Dendritic Cell-Dependent Induction of Regulatory T Cells and Alterations in Gut Microbiota
Source: Front Immunol. 2017 Nov 10;8:1536. doi: 10.3389/fimmu.2017.01536 (PMC5686061; doi:10.3389/fimmu.2017.01536)
Supplement: Supplementary file 2 [file table_2.docx]

**Table S2.** Linear regression analysis of Th2 / Treg and each genera proportion. Results were arranged according to the descending order of coefficient of determination (R^2^).

| **Genus** | **Control** | **Therapeutic group** | |  | **Preventive group** | | **Slope** | **Intercept** | **R^2^** |
| --- | --- | --- | --- | --- | --- | --- | --- | --- | --- |
|  |  | **TM** | **TM+Binf** |  | **TM** | **Binf+TM** |  |  |  |
| Th2/Treg | 1.00 | 1.10 | 0.45 |  | 1.24 | 0.38 | 1.00 | 0.00 | 1.00 |
| *Dorea* | 1.00 | 0.52 | 1.89 |  | 1.06 | 2.48 | -1.81 | 2.90 | 0.82 |
| *Ralstonia* | 1.00 | 1.29 | 0.57 |  | 1.05 | 0.71 | 0.63 | 0.40 | 0.77 |
| *Roseburia* | 1.00 | 0.62 | 1.12 |  | 0.37 | 1.06 | -0.69 | 1.41 | 0.72 |
| *Streptophyta* | 1.00 | 1.31 | 1.64 |  | 1.31 | 1.64 | -0.52 | 1.82 | 0.60 |
| *Sporacetigenium* | 1.00 | 1.23 | 1.72 |  | 0.25 | 1.23 | -1.01 | 1.93 | 0.55 |
| *Bilophila* | 1.00 | 1.21 | 1.26 |  | 1.15 | 2.38 | -0.97 | 2.21 | 0.48 |
| *Coprobacillus* | 1.00 | 0.26 | 16.75 |  | 2.62 | 4.19 | -11.41 | 14.49 | 0.44 |
| *Kandleria* | 1.00 | 3.14 | 5.89 |  | 6.67 | 28.27 | -18.22 | 24.21 | 0.43 |
| *Wolinella* | 1.00 | 0.98 | 0.98 |  | 0.98 | 1.96 | -0.71 | 1.77 | 0.41 |
| *Guggenheimella* | 1.00 | 0.59 | 1.17 |  | 0.72 | 0.86 | -0.37 | 1.17 | 0.40 |
| *Pseudoflavonifractor* | 1.00 | 0.54 | 1.14 |  | 0.47 | 0.75 | -0.45 | 1.15 | 0.38 |
| *Barnesiella* | 1.00 | 1.23 | 0.84 |  | 1.79 | 1.27 | 0.52 | 0.79 | 0.33 |
| *Saccharofermentans* | 1.00 | 1.43 | 2.34 |  | 0.08 | 0.91 | -1.19 | 2.15 | 0.32 |
| *Odoribacter* | 1.00 | 3.10 | 2.14 |  | 2.54 | 0.57 | 1.52 | 0.60 | 0.32 |
| *Clostridium sensu stricto* | 1.00 | 39.26 | 133.49 |  | 1982.68 | 113.86 | 1155.91 | -511.03 | 0.28 |
| *Parabacteroides* | 1.00 | 4.03 | 3.22 |  | 2.30 | 4.74 | -1.95 | 4.68 | 0.27 |
| *Coprococcus* | 1.00 | 0.27 | 0.16 |  | 0.63 | 0.35 | 0.44 | 0.11 | 0.27 |
| *Syntrophococcus* | 1.00 | 0.75 | 0.40 |  | 0.40 | 0.32 | 0.37 | 0.27 | 0.25 |
| *Shuttleworthia* | 1.00 | 0.45 | 4.08 |  | 0.15 | 0.15 | -2.10 | 2.92 | 0.25 |
| *Gordonibacter* | 1.00 | 0.26 | 1.05 |  | 0.26 | 0.52 | -0.49 | 1.02 | 0.25 |
| *Papillibacter* | 1.00 | 1.56 | 0.30 |  | 0.19 | 0.16 | 0.75 | 0.02 | 0.23 |
| *Acidaminobacter* | 1.00 | 8.83 | 24.54 |  | 0.98 | 1.96 | -12.07 | 17.54 | 0.22 |
| *Geosporobacter* | 1.00 | 4.71 | 6.54 |  | 5.23 | 5.50 | -2.36 | 6.56 | 0.19 |
| *Methylophilus* | 1.00 | 2.36 | 3.14 |  | 2.36 | 2.36 | -0.85 | 2.95 | 0.19 |
| *Thermotalea* | 1.00 | 0.73 | 0.48 |  | 0.31 | 1.20 | -0.38 | 1.07 | 0.18 |
| *Bacteroides* | 1.00 | 3.34 | 2.80 |  | 4.43 | 7.34 | -2.47 | 5.85 | 0.17 |
| *Ruminococcus* | 1.00 | 1.31 | 0.33 |  | 0.82 | 1.06 | 0.39 | 0.58 | 0.17 |
| *Rikenella* | 1.00 | 1.41 | 1.49 |  | 1.35 | 0.61 | 0.38 | 0.85 | 0.17 |
| *Clostridium IV* | 1.00 | 0.36 | 0.83 |  | 0.41 | 0.61 | -0.27 | 0.87 | 0.15 |
| *Paraprevotella* | 1.00 | 11.78 | 2.94 |  | 3.44 | 2.45 | 3.95 | 1.02 | 0.13 |
| *Enterorhabdus* | 1.00 | 0.68 | 0.85 |  | 0.68 | 0.34 | 0.23 | 0.52 | 0.13 |
| *Oribacterium* | 1.00 | 0.63 | 2.06 |  | 0.48 | 0.28 | -0.64 | 1.42 | 0.13 |
| *Alistipes* | 1.00 | 1.40 | 1.72 |  | 0.83 | 0.92 | -0.33 | 1.45 | 0.13 |
| *Butyrivibrio* | 1.00 | 1.10 | 1.32 |  | 0.49 | 0.71 | -0.28 | 1.16 | 0.11 |
| *Flavonifractor* | 1.00 | 0.72 | 0.97 |  | 0.24 | 0.52 | -0.25 | 0.90 | 0.10 |
| *Johnsonella* | 1.00 | 0.29 | 0.15 |  | 0.15 | 0.15 | 0.29 | 0.10 | 0.09 |
| *Allobaculum* | 1.00 | 1.07 | 6.54 |  | 6.07 | 3.57 | -1.97 | 5.29 | 0.09 |
| *Butyricicoccus* | 1.00 | 0.05 | 0.61 |  | 0.37 | 0.56 | -0.25 | 0.73 | 0.08 |
| *Anaeroplasma* | 1.00 | 39.55 | 24.57 |  | 4.80 | 22.39 | -11.26 | 27.87 | 0.08 |
| *Turicibacter* | 1.00 | 0.17 | 0.04 |  | 0.04 | 0.10 | 0.27 | 0.04 | 0.07 |
| *Desulfitibacter* | 1.00 | 0.22 | 0.55 |  | 0.11 | 0.44 | -0.23 | 0.65 | 0.07 |
| *Vampirovibrio* | 1.00 | 0.65 | 0.47 |  | 0.35 | 0.43 | 0.17 | 0.44 | 0.06 |
| *Sporanaerobacter* | 1.00 | 0.98 | 0.98 |  | 0.49 | 0.74 | -0.14 | 0.95 | 0.06 |
| *Clostridium XlVb* | 1.00 | 0.21 | 0.40 |  | 0.34 | 0.73 | -0.20 | 0.70 | 0.06 |
| *Ruminococcus2* | 1.00 | 0.27 | 1.23 |  | 0.55 | 0.32 | -0.26 | 0.89 | 0.06 |
| *Meniscus* | 1.00 | 1.18 | 0.39 |  | 0.39 | 0.79 | 0.21 | 0.57 | 0.06 |
| *Peptococcus* | 1.00 | 0.30 | 0.30 |  | 0.30 | 0.30 | 0.19 | 0.29 | 0.05 |
| *Lactobacillus* | 1.00 | 0.57 | 2.36 |  | 2.29 | 1.38 | -0.46 | 1.91 | 0.05 |
| *Tannerella* | 1.00 | 0.61 | 0.25 |  | 0.78 | 0.97 | 0.17 | 0.58 | 0.05 |
| *Hespellia* | 1.00 | 0.12 | 1.78 |  | 1.19 | 0.48 | -0.36 | 1.22 | 0.05 |
| *Moryella* | 1.00 | 0.98 | 0.49 |  | 0.25 | 0.49 | 0.18 | 0.49 | 0.05 |
| *Marvinbryantia* | 1.00 | 0.12 | 0.74 |  | 0.11 | 0.20 | -0.22 | 0.61 | 0.04 |
| *Lactonifactor* | 1.00 | 0.05 | 0.09 |  | 0.33 | 0.93 | -0.24 | 0.68 | 0.04 |
| *Erysipelotrichaceae* | 1.00 | 0.55 | 1.27 |  | 0.42 | 0.26 | -0.20 | 0.87 | 0.04 |
| *Hydrogenoanaerobacterium* | 1.00 | 1.06 | 1.00 |  | 0.27 | 0.60 | -0.16 | 0.92 | 0.03 |
| *Dethiosulfatibacter* | 1.00 | 0.44 | 0.65 |  | 0.22 | 0.44 | -0.13 | 0.66 | 0.03 |
| *Anaerosalibacter* | 1.00 | 1.82 | 0.88 |  | 0.10 | 0.39 | 0.28 | 0.60 | 0.03 |
| *Paraeggerthella* | 1.00 | 0.49 | 0.98 |  | 0.25 | 0.25 | -0.15 | 0.72 | 0.03 |
| *Defluviitalea* | 1.00 | 0.27 | 0.32 |  | 0.10 | 0.14 | 0.15 | 0.24 | 0.03 |
| *Saccharibacteria* | 1.00 | 0.20 | 0.84 |  | 0.44 | 0.35 | -0.14 | 0.68 | 0.03 |
| *Anaerosporobacter* | 1.00 | 0.98 | 1.78 |  | 1.27 | 0.29 | 0.20 | 0.90 | 0.02 |
| *Anaerovibrio* | 1.00 | 3.31 | 1.03 |  | 0.41 | 1.24 | 0.41 | 1.06 | 0.02 |
| *Prevotella* | 1.00 | 9.38 | 5.89 |  | 3.71 | 2.84 | 0.98 | 3.74 | 0.01 |
| *Lachnoanaerobaculum* | 1.00 | 0.04 | 0.30 |  | 0.07 | 0.04 | 0.11 | 0.19 | 0.01 |
| *Cellulosilyticum* | 1.00 | 0.01 | 0.08 |  | 0.04 | 0.20 | 0.10 | 0.18 | 0.01 |
| *Acetivibrio* | 1.00 | 0.10 | 0.22 |  | 0.10 | 0.20 | 0.09 | 0.25 | 0.01 |
| *Anaerostipes* | 1.00 | 0.57 | 0.14 |  | 0.24 | 0.91 | -0.08 | 0.64 | 0.01 |
| *Sediminibacterium* | 1.00 | 6.54 | 5.23 |  | 5.23 | 3.93 | 0.39 | 4.06 | 0.01 |
| *Helicobacter* | 1.00 | 0.45 | 0.33 |  | 0.35 | 0.60 | 0.05 | 0.51 | 0.00 |
| *Alkalitalea* | 1.00 | 1.19 | 1.41 |  | 1.04 | 0.74 | 0.04 | 1.04 | 0.00 |
| *Clostridium XlVa* | 1.00 | 0.41 | 0.79 |  | 0.26 | 0.23 | -0.05 | 0.58 | 0.00 |
| *Mucispirillum* | 1.00 | 0.60 | 0.63 |  | 0.34 | 0.52 | -0.03 | 0.65 | 0.00 |
| *Anaerotruncus* | 1.00 | 0.36 | 0.31 |  | 0.32 | 0.55 | 0.04 | 0.48 | 0.00 |
| *Fastidiosipila* | 1.00 | 21.20 | 12.56 |  | 2.36 | 5.50 | -0.95 | 9.31 | 0.00 |
| *Paralactobacillus* | 1.00 | 0.79 | 3.14 |  | 3.14 | 0.79 | 0.14 | 1.66 | 0.00 |
| *Alloprevotella* | 1.00 | 1.55 | 3.86 |  | 3.12 | 0.97 | -0.14 | 2.22 | 0.00 |
| *Dongia* | 1.00 | 25.59 | 0.44 |  | 1.02 | 14.83 | 0.85 | 7.87 | 0.00 |
| *Anaerovorax* | 1.00 | 11.27 | 7.34 |  | 1.11 | 2.13 | -0.32 | 4.83 | 0.00 |
| *Robinsoniella* | 1.00 | 0.75 | 0.83 |  | 0.23 | 0.28 | 0.02 | 0.60 | 0.00 |
| *Lachnospiracea* | 1.00 | 0.12 | 0.42 |  | 0.18 | 0.22 | 0.02 | 0.37 | 0.00 |
| *Oscillibacter* | 1.00 | 0.68 | 0.89 |  | 0.39 | 0.37 | -0.01 | 0.67 | 0.00 |
