# Supplementary figures and images for: Bifidobacterium infantis Potentially Alleviates Shrimp Tropomyosin-Induced Allergy by Tolerogenic Dendritic Cell-Dependent Induction of Regulatory T Cells and Alterations in Gut Microbiota
Source: Front Immunol. 2017 Nov 10;8:1536. doi: 10.3389/fimmu.2017.01536 (PMC5686061; doi:10.3389/fimmu.2017.01536)

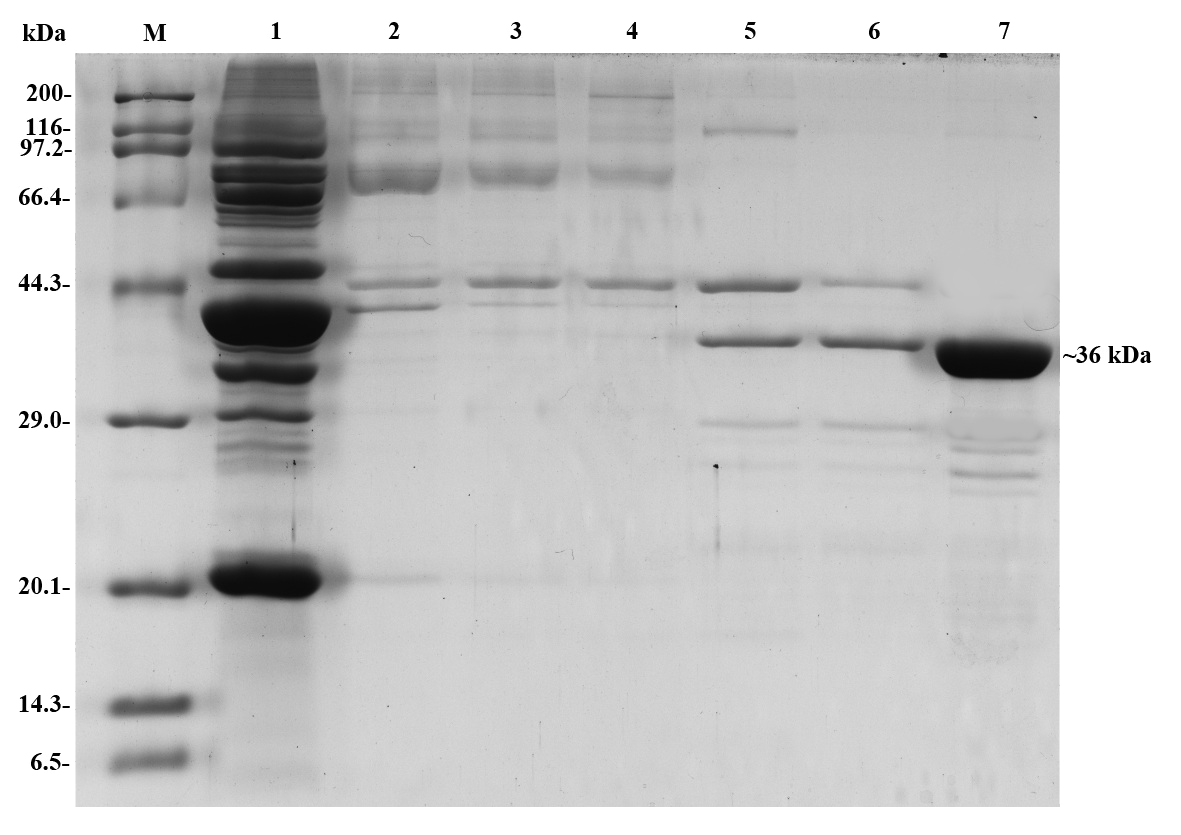

Supplement: Figure S1 — Shrimp tropomyosin (Tm) purification from Litopenaeus vannamei. Protein products from each purification step were detected by SDS-PAGE and coomassie blue staining. Lanes 1–4, the supernatant of each extracting procedure by Buffer A; lane 5, the supernatant of extracting procedure by Buffer B; lane 6, the supernatant after heat treatment; lane 7, the final Tm product. [file image_1.jpeg]

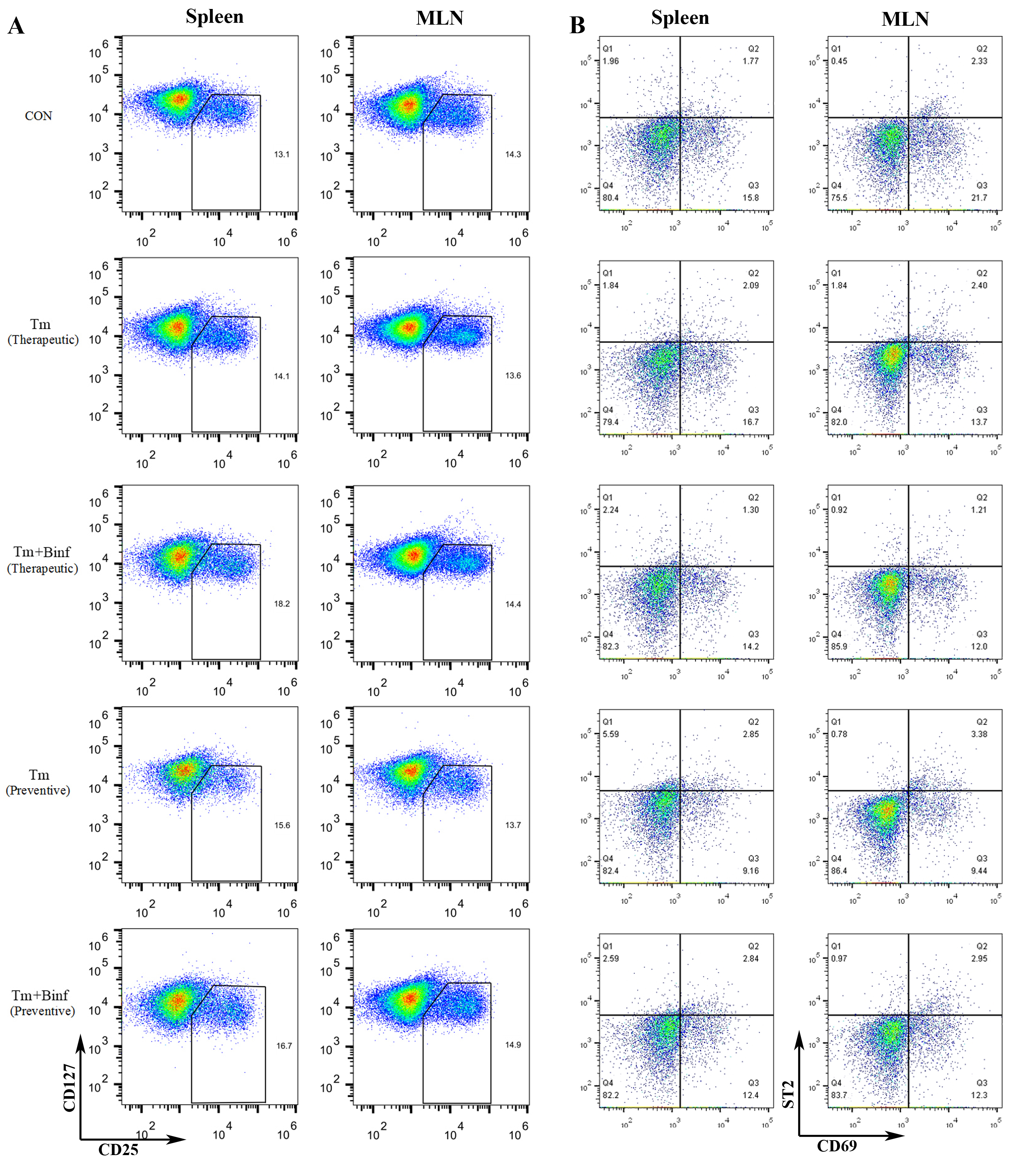

Supplement: Figure S2 — Binf modulates Th2 and regulatory T cell (Treg) proportion in mesenteric lymph node (MLN) and spleen. (A) Flow cytometry results for Treg (CD4+CD25+CD127low/−) in spleen and MLN. (B) Flow cytometry results for Th2 (CD69+ST2+) in spleen and MLN. [file image_2.jpeg]

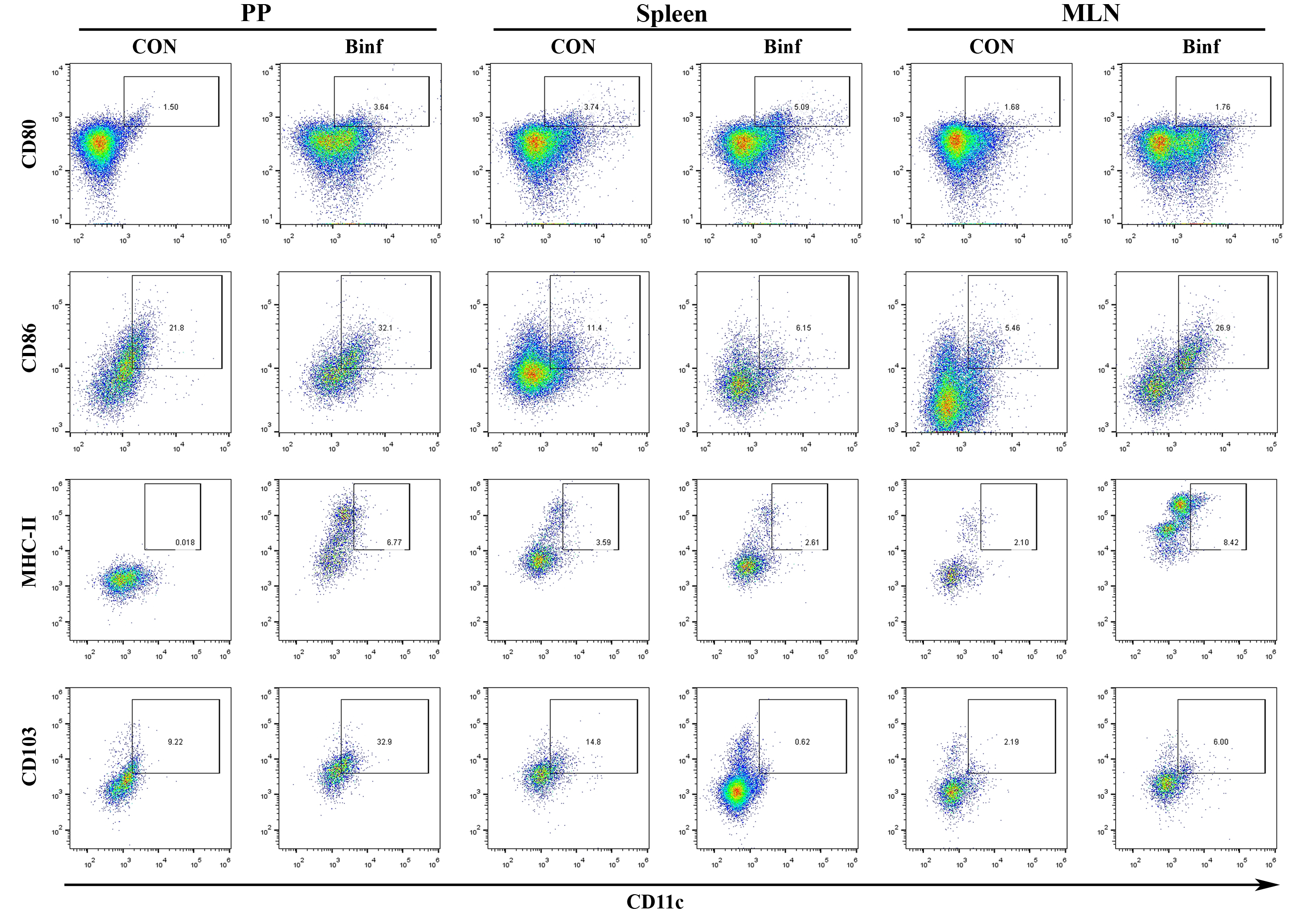

Supplement: Figure S3 — Binf promotes dendritic cell (DC) maturation and induces CD103+ DC. CD80+, CD86+, MHC-II+, and CD103+ DCs (CD11c+) proportion in each groups was measured by flow cytometry. [file image_3.jpeg]

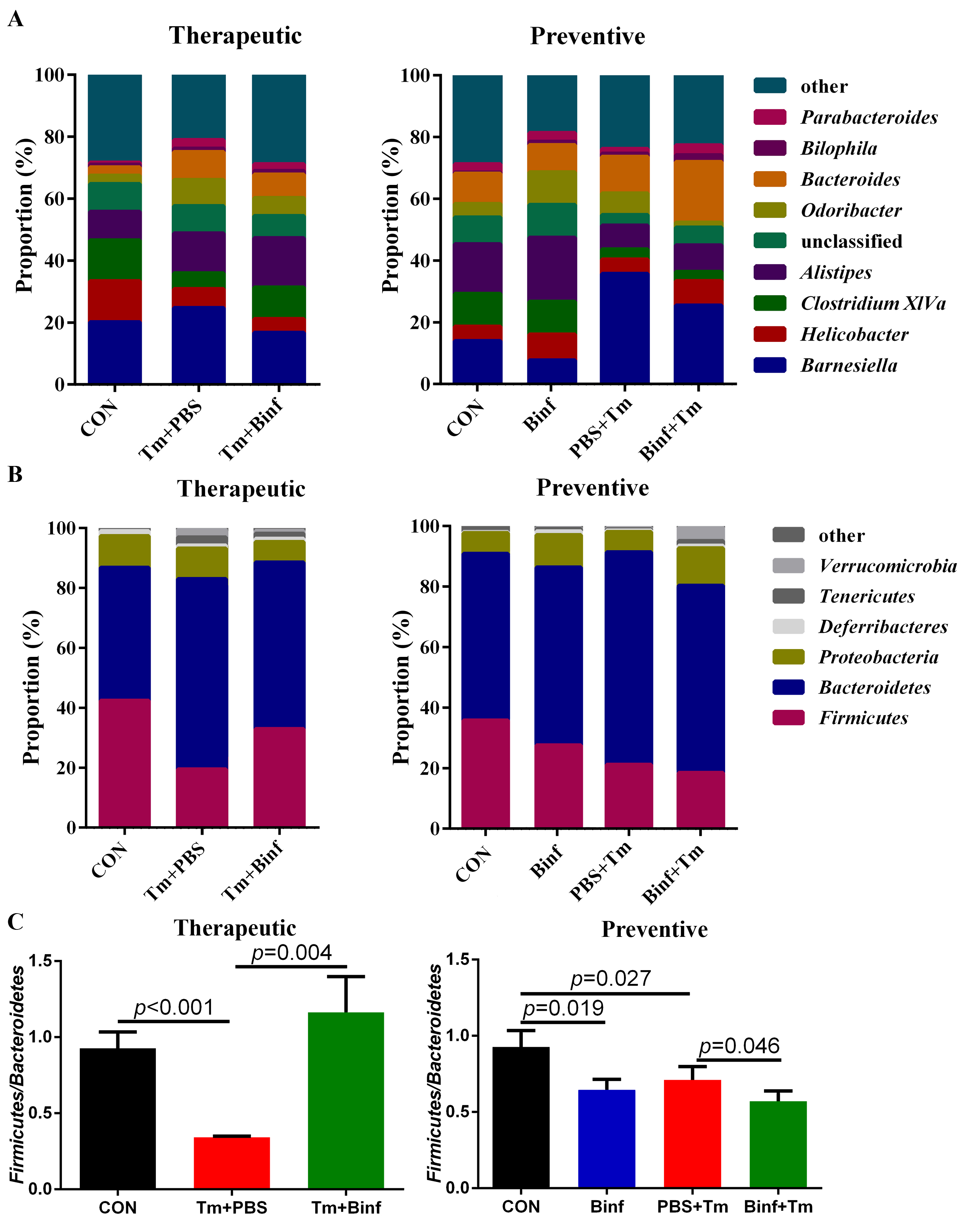

Supplement: Figure S4 — Effect of Binf on fecal microbiota composition in tropomyosin (Tm)-sensitized mice. (A) Sequencing analysis of fecal microbiota composition from mice at genera level. (B) Sequencing analysis of fecal microbiota composition from mice at phyla level. (C) The ratio of two most abundant phyla (Firmicutes/Bacteroidetes) in each group. [file image_4.jpeg]
